# Case Report: Value of MR relaxometry and spectroscopy in multimodal imaging characterization of limbic encephalitis in systemic sclerosis

**DOI:** 10.3389/fradi.2026.1945816

**Published:** 2026-09-30

**Authors:** Florian Awin Stolz, Andrei Manzhurtsev, Marlene Bönstrup, Daniel Groener, Nico Melzer, Felix Rosenow, Elke Hattingen, Christophe Théo Arendt

**Affiliations:** 1 Institute of Neuroradiology, Goethe University Frankfurt, University Hospital, Frankfurt, Germany; 2 Department of Neurology, Goethe University Frankfurt, University Hospital, Frankfurt, Germany; 3 Nuclear Medicine, Clinic for Radiology and Nuclear Medicine, Goethe University Frankfurt, University Hospital, Frankfurt, Germany; 4 Department of Neurology, Medical Faculty and University Hospital Düsseldorf, Heinrich-Heine University of Düsseldorf, Düsseldorf, Germany; 5 Epilepsy Center Frankfurt Rhine-Main, Center of Neurology and Neurosurgery, Goethe University Frankfurt, University Hospital, Frankfurt, Germany

**Keywords:** autoantibodies, limbic encephalitis, magnetic resonance spectroscopy, N-acetylaspartate, positron emission tomography computed tomography, scleroderma, systemic

## Abstract

Central nervous system involvement is uncommon in systemic sclerosis, and inflammatory temporomesial syndromes in this context are rarely reported. We present a 58-year-old man with systemic sclerosis who developed subacute cognitive impairment and dialeptic seizures arising from the right temporal lobe. Video electroencephalography confirmed a right temporal seizure onset zone. Brain magnetic resonance imaging demonstrated enlargement and subtle T2 hyperintensity of the right hippocampus and amygdala, while fluorodeoxyglucose-positron emission tomography/computed tomography images showed focal hypermetabolism of the enlarged right amygdala. Proton magnetic resonance spectroscopy demonstrated bilateral reduction of total N-acetylaspartate compared with an age-matched healthy reference subject, with a more pronounced difference on the right, a pattern compatible with bilateral, right-accentuated neuronal dysfunction or injury. MR relaxometry demonstrated significant bilateral T2 prolongation in the amygdalae and hippocampal heads, whereas T1 abnormalities were more region-dependent, involving the right amygdala and both hippocampal heads. Serum testing detected anti-pyruvate dehydrogenase antibodies, whereas routine clinical neural antibody testing was negative. The patient became seizure-free under antiseizure medication and immunosuppressive therapy with cyclophosphamide. Notably, although the unilateral conventional imaging findings did not meet the bilateral mesial temporal criterion required by current consensus criteria for definite autoimmune limbic encephalitis, MR relaxometry revealed bilateral temporomesial abnormalities. This finding is consistent with available neuropathological and experimental evidence and suggests that routine imaging may underestimate disease extent. This case highlights a rare presentation of antibody-atypical limbic encephalitis in the setting of systemic sclerosis and illustrates how multimodal imaging can support diagnosis and tissue characterization when routine antibody testing is unrevealing.

## 1 Introduction

Autoimmune limbic encephalitis (LE) is an inflammatory disorder of the central nervous system that typically involves temporomesial structures and presents with subacute cognitive impairment, altered mental status, and seizures (1). Diagnosis can be particularly challenging when established neural-autoantibody panels are negative and conventional structural magnetic resonance imaging (MRI) findings are subtle or unilateral, as the clinical picture then overlaps with epileptic, infectious, vascular, toxic-metabolic, and neurodegenerative disorders (2). Current consensus criteria require bilateral mesial temporal abnormality on conventional MRI for a diagnosis of definite autoimmune LE, whereas unilateral imaging findings are instead classified under the broader, less specific category of possible autoimmune encephalitis (1). Consequently, patients with clinically suspected LE but incomplete conventional imaging findings may remain diagnostically uncertain.

Central nervous system involvement is uncommon in systemic sclerosis (SSc) and is more often attributed to vasculopathy than to parenchymal inflammation, although rare inflammatory and encephalitic syndromes, including LE, have been described (3–5). This distinction may be especially difficult in antibody-negative or serologically atypical presentations. Moreover, neuropathological studies have demonstrated that limbic inflammation may be bilateral even when abnormalities on conventional MRI are subtle or apparently unilateral (6, 7). Quantitative MRI techniques may therefore provide complementary information on tissue alterations that are not readily apparent on conventional imaging. In particular, T2 relaxometry has previously been proposed as a potentially useful adjunct in patients with suspected LE and epilepsy (8). Proton MR spectroscopy (MRS) offers a complementary metabolic perspective by assessing regional alterations in neurometabolites that may accompany inflammatory tissue changes even when structural abnormalities are limited (9).

In this report, we describe a patient with SSc and clinically suspected antibody-atypical LE to investigate whether quantitative MRI can identify mesial temporal tissue abnormalities beyond those visible on conventional MRI, including possible contralateral involvement, and whether MRS can provide complementary evidence of regional metabolic involvement. We further examine whether integrating quantitative MRI and MRS with conventional MRI, fluorodeoxyglucose-positron emission tomography/computed tomography (FDG-PET/CT), and electroencephalography (EEG) can improve the characterization of an otherwise diagnostically challenging presentation.

## 2 Case presentation

A 58-year-old man with SSc presented with cognitive symptoms including confusion, increasing forgetfulness, episodes of mental absence, and spatial disorientation. According to the patient's history, the most recent known seizure before the study MRI had occurred approximately one month earlier. Prolonged video-EEG monitoring was initiated in late January 2026 and continued over several days, ending one day before the study MRI in February 2026. It demonstrated rhythmic right temporal slowing with occasional sharp waves, but no electrographic seizures.

The clinical timeline for this patient is summarized in Table 1. Antiseizure medication was initiated with levetiracetam (1,000 mg twice daily), which was subsequently switched due to side effects and replaced by lamotrigine (100 mg twice daily) and brivaracetam (100 mg twice daily). In interdisciplinary consultation with rheumatology, immunosuppressive therapy was escalated to cyclophosphamide. The patient subsequently remained seizure-free under combined antiseizure and immunosuppressive treatment, which was well tolerated. At the most recent follow-up, he reported subjective improvement in his cognitive symptoms and good overall well-being and had returned to work after a period of illness-related work incapacity. Detailed treatment regimens and doses are provided in Table 1.

**Table 1:** Clinical timeline of the patient: key events, findings, and procedures from symptom onset to last follow-up.

Date	Event	Key findings/procedures
February 2025	First manifestation of systemic sclerosis	Raynaud phenomenon; subsequently accompanied by puffy fingers and rheumatoid arthritis, with an active pattern on nailfold capillaroscopy; treatment with methotrexate (15 mg s.c. once weekly) was initiated
November 2025	Neurological symptom onset	Subacute cognitive decline: confusion, increasing forgetfulness, episodes of impaired awareness, and spatial disorientation
December 2025	Initial outpatient MRI	Brain MRI performed on an outpatient basis and reported as normal; levetiracetam initiated (1,000 mg twice daily)
January 2026	Admission and diagnostic work-up	Unremarkable neurological examination; right temporal seizure onset; no established neural antibodies in serum or CSF; 1 leukocyte/µL; protein 590 mg/L; oligoclonal bands type 4; anti-pyruvate dehydrogenase antibodies positive in serum
February 2026	Multimodal MRI, FDG-PET/CT and treatment escalation	Conventional MRI, MR relaxometry (T1/T2), ^1^H-MR spectroscopy, FDG-PET/CT with MRI fusion. Switch to lamotrigine (100 mg twice daily) and brivaracetam (100 mg twice daily); cyclophosphamide (1,000 mg i.v., four cycles at 2-week intervals), prednisolone (10 mg/day) and methotrexate (15 mg s.c)
April 2026	Last seizure of active phase, initiation of rituximab	Seizure-free thereafter; rituximab initiated as on-label therapy for rheumatoid arthritis (1,000 mg i.v. × 2, two weeks apart) with continued methotrexate and prednisolone (7,5 mg)
June 2026	MRI with HARNESS protocol and EEG Follow-up	EEG demonstrated an irritative zone maximal at T4, without recorded seizures. Follow-up structural MRI showed stable findings without interval change, while quantitative MRI and MR spectroscopy could not be repeated.

## 3 Multimodal imaging

The patient's brain MRI was performed according to a Harmonized Neuroimaging of Epilepsy Structural Sequences (HARNESS) epilepsy protocol, including a 3D isotropic T2-weighted fluid-attenuated inversion recovery (FLAIR) sequence with 1 mm^3^ voxel size (10). MRI demonstrated marked enlargement and mildly increased T2-FLAIR signal intensity of the right hippocampus and amygdala (Figure 1). The left hippocampus also presented with questionable rostral enlargement, and additional involvement in the LE process could not be excluded. Diffusion-weighted imaging (DWI) with apparent diffusion coefficient maps and susceptibility-weighted imaging were unremarkable.

**Figure 1:**
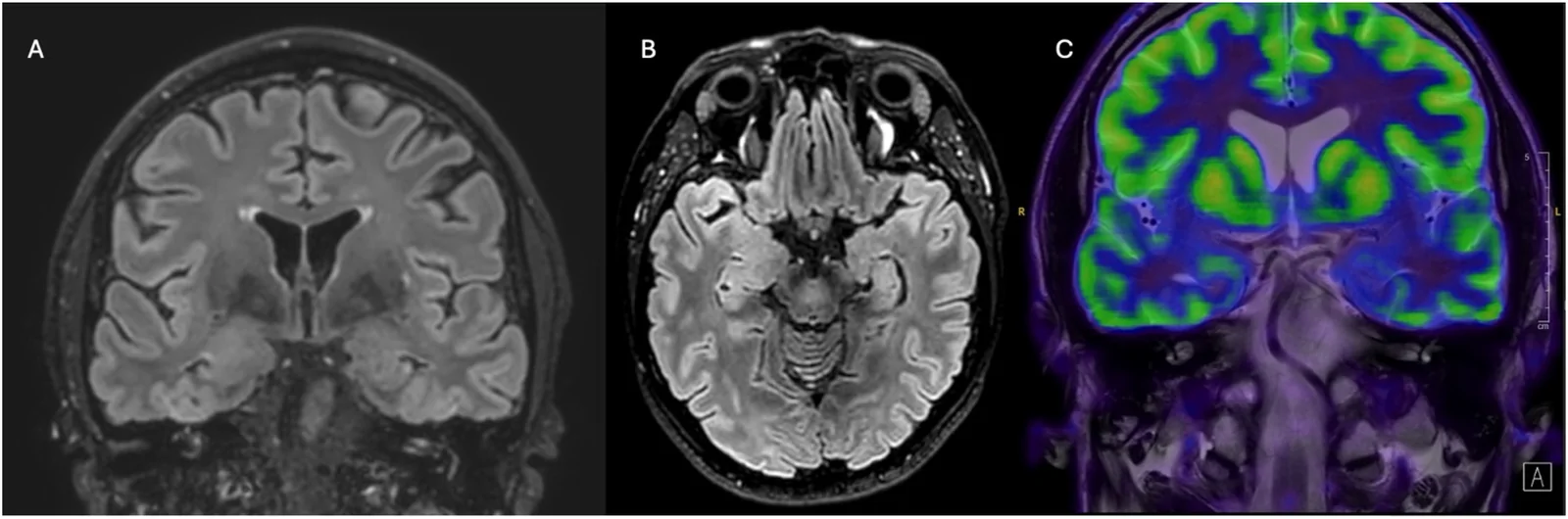
Conventional MRI and PET-CT findings of right-dominant limbic encephalitis. **(A)** Coronal and **(B)** axial T2-weighted FLAIR images show enlargement and subtle hyperintensity of the right hippocampus and amygdala. **(C)** Fused FDG-PET/CT-MRI image (fluorodeoxyglucose-positron emission tomography/computed tomography shows focal relative hypermetabolism of the enlarged right amygdala. FDG, fluorodeoxyglucose; PET, positron emission tomography; CT, computed tomography; MRI, magnetic resonance imaging; FLAIR, fluid-attenuated inversion recovery.

FDG-PET/CT of the neurocranium demonstrated relatively increased glucose utilization in the enlarged and MRI-abnormal right amygdala, while glucose utilization in the remaining neurocranium was physiological (Figure 1). PET/CT-MRI fusion images confirmed relative focal hypermetabolism of the right amygdala, compatible with the suspected diagnosis of LE.

A multiparametric quantitative MR protocol was applied to further characterize tissue properties within the temporomesial regions (11). T1 and T2 values were extracted from manually placed regions of interest (ROI) in the right and left amygdala and the right and left hippocampal head (Figure 2). To assess intra-rater reliability, ROI placement was repeated by the same rater after an interval of more than four weeks. The rater was aware of patient/reference status at both assessments. Measurements from the first assessment were used for the primary single-case analysis. Intra-rater reliability was evaluated separately for each ROI using a two-way mixed effects intraclass correlation coefficient (ICC) for absolute agreement and single measurements. ROI-specific ICCs are reported in Table 2. To account for inter-individual and inter-hemispheric variability, the patient's regional quantitative values were compared with the corresponding hemisphere-specific distributions of healthy reference subjects. The qT1 reference group included seven subjects (six male, one female; mean age 53.7 ± 10.3 years), five of whom also constituted the qT2 reference group (four male, one female; mean age 52.6 ± 12.3 years). Like the patient, all reference subjects were right-handed, and the patient's age (58 years) fell within the age distribution of both reference groups. All participants underwent imaging on the same 3-Tesla MR system (MR 7700; Philips Healthcare, Best, The Netherlands) using the same respective quantitative protocols as the patient. Quantitative T1 mapping was performed using a 3D fast field echo (FFE) acquisition with variable flip angles of 5° and 32° (repetition time [TR] = 30 ms, echo time [TE] = 3.2–25.7 ms with 4.5-ms echo spacing; acquired voxel size = 1.2 × 1.6 × 1.2 mm^3^; reconstructed voxel size = 1.0 × 1.0 × 1.0 mm^3^). Quantitative T2 mapping was performed using a coronal multi-echo 2D turbo spin-echo (TSE) acquisition with nine echoes spanning 12–96 ms (TR = 3,000 ms, flip angle = 90°, slice thickness = 5 mm, interslice spacing = 5.5 mm). Single-case inference was performed separately for each ROI using the two-sided Crawford–Howell modified *t*-test, with Holm correction applied jointly across all eight regional qT1 and qT2 comparisons. Full ROI-specific statistics are provided in Table 2.

**Figure 2:**
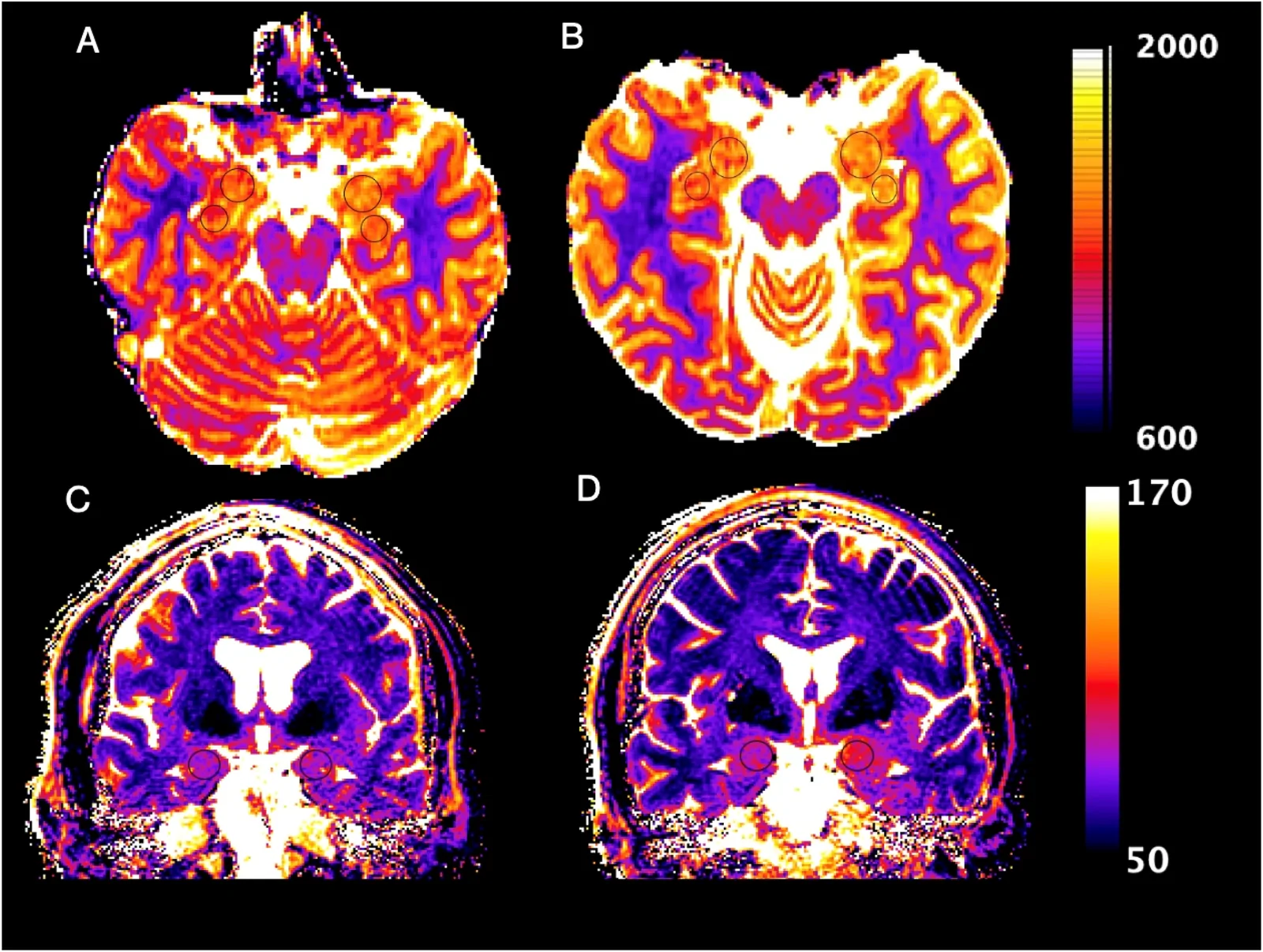
Representative quantitative T1 and T2 maps of a healthy reference subject and the patient. **(A,B)** Axial quantitative T1 maps of a healthy reference subject **(A)** and the patient **(B) (C,D)** Coronal quantitative T2 maps at the level of the amygdalae in a healthy reference subject **(C)** and the patient **(D)** Manually placed regions of interest are shown bilaterally in the amygdalae and hippocampal heads. The respective color scales indicate quantitative T1 and T2 relaxation times in milliseconds.

**Table 2:** Single-case comparison of regional quantitative T1 and T2 relaxation values with healthy reference distributions.

Parameter	Region	*n*	Patient ROI mean ± voxel SD (ms)	Reference mean ± between-subject SD (ms)	ICC [95%CI]	zCC	CH t	df	*p* (Holm-adjusted)
qT1	Right amygdala	7	1,581 ± 143	1,370.1 ± 28.9	0.88 [0.65–0.97]	7.299	6.828	6	0.004**
qT1	Left amygdala	7	1,472 ± 101	1,415.1 ± 35.0	0.9 [0.58–0.98]	1.626	1.521	6	0.179
qT1	Right hippocampal head	7	1,546 ± 63	1,317.0 ± 63.7	0.89 [0.64–0.98]	3.594	3.362	6	0.048*
qT1	Left hippocampal head	7	1,506 ± 107	1,371.6 ± 27.7	0.92 [0.89–0.96]	4.85	4.537	6	0.020*
qT2	Right amygdala	5	103 ± 5	82.0 ± 4.6	0.92 [0.57–0.98]	4.583	4.183	4	0.048*
qT2	Left amygdala	5	109 ± 3	87.4 ± 3.0	0.94 [0.65–0.99]	7.281	6.647	4	0.016*
qT2	Right hippocampal head	5	109 ± 5	83.8 ± 2.7	0.93 [0.65–0.99]	9.391	8.573	4	0.0077**
qT2	Left hippocampal head	5	105 ± 7	87.6 ± 3.6	0.87 [0.41–0.98]	4.771	4.355	4	0.048*

Patient values are reported as region of interest (ROI) mean ± within-ROI voxel standard deviation (SD). Reference values are reported as mean ± between-subject standard deviation (SD) of subject-level ROI means. The patient's voxel SD is descriptive and was not used for single-case inference. Patient ROI means were compared with the corresponding hemisphere-specific healthy reference distributions using the two-sided Crawford–Howell modified *t*-test. Holm correction was applied jointly across all eight regional qT1 and qT2 comparisons. Intra-rater reliability was assessed separately for each ROI using a two-way mixed-effects intraclass correlation coefficient (ICC) for absolute agreement and single measurements. ICCs are reported with their 95% confidence intervals (CIs). n, number of healthy reference subjects; zCC, standardized difference between the patient's ROI mean and the reference mean; CH t, Crawford–Howell modified t-statistic; df, degrees of freedom. Statistical significance was determined based on Holm-adjusted *p*-values: *p < 0.05, **p < 0.01, ***p < 0.001.

Compared with the healthy reference distributions, qT1 values were significantly increased in the right amygdala and in both hippocampal heads, whereas the left amygdala did not show a statistically significant qT1 abnormality. In contrast, qT2 values were significantly increased in all four analyzed temporomesial regions, with all differences remaining significant after Holm correction (Table 2).

Overall, quantitative mapping demonstrated bilateral temporomesial relaxation abnormalities, with a more widespread pattern for qT2 than for qT1. These findings provide additional quantitative information complementary to structural MRI and FDG-PET/CT.

MRS was performed using a point-resolved spectroscopy (PRESS) sequence with a 30 × 15 × 10 mm^3^ temporomesial voxel positioned to include the hippocampal and amygdalar regions (Figure 3). Acquisition parameters were TR = 2,000 ms, TE = 32 ms, 160 averages, 1,024 spectral points, and a spectral bandwidth of 2,000 Hz, with a total acquisition time of 5 min 31 s. Variable power RF pulses with optimized relaxation delays (VAPOR) water suppression was applied with a water-suppression bandwidth of 130 Hz. In addition, four unsuppressed water-reference scans were acquired using the same TR and TE. Absolute metabolite concentrations were calculated using water-referenced scaling with correction for cerebrospinal fluid (CSF) fraction and relaxation effects. For relaxation correction, voxel-specific quantitative MRI-derived water T1 and T2 values were used. Spectral fitting was performed using Osprey, an open-source magnetic resonance spectroscopy analysis toolbox (12). Although MRS data had also been acquired in additional subjects from the qT1 and qT2 reference groups, their spectral quality was insufficient for reliable metabolite quantification. The MRS comparison was therefore limited to a single age-matched healthy reference subject with corresponding bilateral temporomesial voxel placement. Spectral quality metrics were comparable between the patient and the healthy reference subject, with signal-to-noise (SNR)/ full width at half maximum (FWHM) values of 25.4/6.8 Hz (left) and 35.4/8.2 Hz (right) in the patient, compared with 21.4/7.9 Hz (left) and 18.8/8.4 Hz (right) in the healthy reference subject. In addition to the left-right comparison, metabolite concentrations were compared with those obtained from an age-matched healthy reference subject using corresponding bilateral temporomesial voxel placement. The most pronounced difference was observed for total N-acetylaspartate (tNAA), which was reduced bilaterally in the patient compared with the healthy reference subject, with a more pronounced reduction on the right side. The most consistent finding was a bilateral reduction in tNAA, with a greater difference on the right (−25.9%) than on the left (−10.4%). Right-sided tCho was also lower than in the healthy reference subject (−21.0%), whereas left-sided tCho was comparable. The remaining metabolite concentrations are summarized in Table 3. Thus, MRS demonstrated a bilateral reduction in tNAA with a clear right-sided predominance, accompanied by an additional right-sided reduction in tCho.

**Figure 3:**
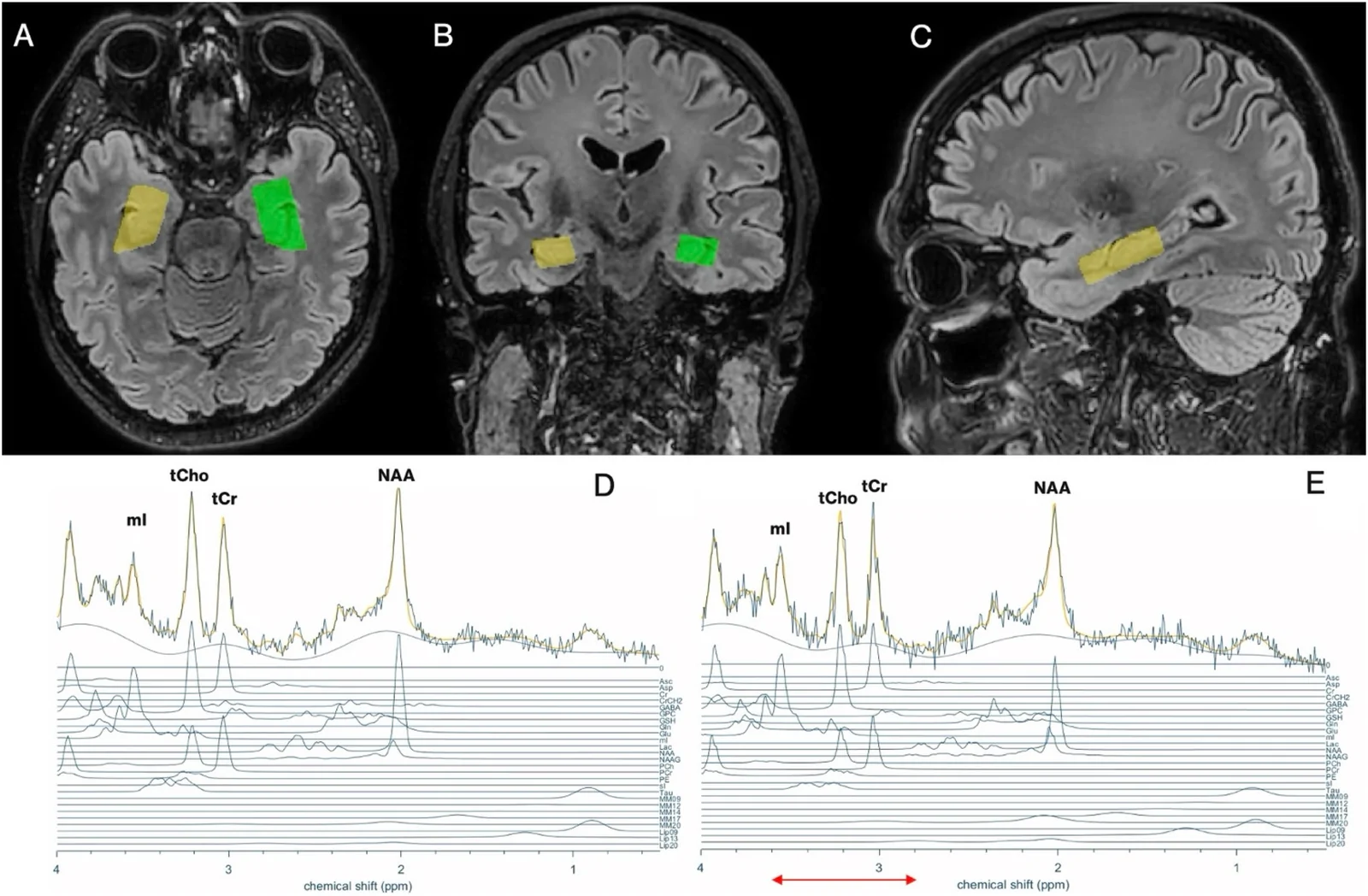
Single-voxel proton MR spectroscopy of the temporomesial region. **(A)** transversal, **(B)** coronal, and **(C)** sagittal FLAIR images demonstrate placement of the spectroscopy voxel including the hippocampus and amygdala. **(D)** Left-sided and **(E)** right-sided single-voxel proton MR spectra fitted in Osprey software, shown with corresponding spectral fits and metabolite basis spectra. In panel E, the red double-headed arrow marks the spectral region in which the residual out-of-voxel artifact may have influenced metabolite quantification. FLAIR, fluid-attenuated inversion recovery; ppm, parts per million; mI, myo-inositol; tCho, total choline; tCr, total creatine; NAA, N-acetylaspartate.

**Table 3:** Relaxation-corrected metabolite concentrations in the patient and an age-matched healthy reference subject.

Metabolite	Side	Patient concentration (mmol/L)	Patient CRLB	Healthy reference concentration (mmol/L)	Healthy reference CRLB
tNAA	Right	12.0	7%	16.2	6%
	Left	14.3	6%	16.0	7%
tCho	Right	3.8	4%	4.8	4%
	Left	4.5	3%	4.7	4%
tCr	Right	12.8	4%	14.2	4%
	Left	13.8	3%	13.8	5%

Water-referenced and relaxation-corrected metabolite concentrations obtained from corresponding bilateral temporomesial voxels are shown for the patient and a single age-matched healthy reference subject. Relative Cramér–Rao lower bounds (CRLBs; %SD) were obtained from the LCModel fits and are reported as measures of fitting uncertainty.

tNAA, total N-acetylaspartate; tCho, total choline; tCr, total creatine; mI,.

## 4 Discussion

This case illustrates the diagnostic complexity of LE in a patient with SSc and an atypical autoantibody profile. The diagnosis was supported by the convergence of clinical, electrophysiological, metabolic, and structural findings: the patient developed subacute cognitive impairment and focal seizures, MRI demonstrated enlargement and signal abnormality of the right hippocampus and amygdala, FDG-PET/CT showed focal hypermetabolism of the right amygdala, and video-EEG confirmed a right temporal seizure onset zone. This multimodal topographic agreement argues against an incidental imaging abnormality and supports a right-dominant temporomesial inflammatory and epileptogenic process. The imaging pattern also argues against isolated hippocampal sclerosis as the sole explanation for the findings. Although hippocampal sclerosis may be associated with hippocampal T2/FLAIR signal abnormality and chronic epileptogenicity, the marked volume increase of the right hippocampus together with involvement of the amygdala favored an active limbic process. In particular, amygdalar enlargement and focal amygdalar hypermetabolism on FDG-PET/MRI are more consistent with inflammatory limbic involvement than with a purely chronic hippocampal sclerosis pattern (7). In addition, low-grade neoplasm and peri-ictal changes were considered as relevant differential diagnoses. The absence of progressive structural changes on follow-up MRI and of a choline-dominant spectroscopic pattern argued against a progressive neoplastic process, although an indolent low-grade glioma cannot be definitively excluded without histopathological confirmation (13, 14). Notably, the outpatient MRI obtained in December 2025 had been reported as normal, whereas the February 2026 MRI demonstrated marked right mesial temporal enlargement and signal abnormalities, indicating radiological evolution over a relatively short interval and supporting an acquired, evolving process rather than a longstanding structural abnormality. Follow-up structural MRI in June 2026 showed no interval change despite seizure freedom and clinical improvement. This radiological stability does not necessarily contradict an inflammatory interpretation, as structural MRI abnormalities in autoimmune encephalitis may persist despite clinical improvement and do not invariably normalize after treatment (15). Quantitative MRI and MR spectroscopy could not be repeated at follow-up, precluding assessment of whether the previously observed quantitative relaxation and metabolic abnormalities changed over time. An acute peri-ictal explanation was considered less likely given the approximately one-month interval since the last clinically reported seizure, absence of electrographic seizures during video-EEG monitoring completed one day before MRI, and unremarkable DWI/ADC maps (16). However, subclinical or longer-lasting seizure-related contributions cannot be excluded.

The quantitative MR findings further support the presence of bilateral mesial temporal tissue alterations, although their interpretation should remain cautious. T2 values were significantly prolonged in all four analyzed regions, including the bilateral amygdalae and hippocampal heads, whereas T1 abnormalities were more region-dependent, reaching significance in the right amygdala and both hippocampal heads but not in the left amygdala. Thus, the bilateral pattern was considerably more consistent for T2 than for T1, which showed a more right-accentuated distribution. Increased T2 values in particular have been reported as a potential quantitative imaging marker in LE, probably reflecting inflammatory tissue changes due to increased water content (8).

Notably, qT2 was higher in the left than in the right amygdala despite the otherwise predominantly right-sided abnormalities on EEG, structural MRI, FDG-PET, and MRS. In suspected LE, qT2 abnormalities have been reported without significant hemispheric lateralization (8), while quantitative imaging studies indicate particularly pronounced involvement of the amygdala (7), suggesting that regional qT2 magnitude may not directly reflect the side of the clinically dominant process. Conversely, Zhang et al. (17) demonstrated that hippocampal qT2 can contribute to lateralization of the epileptogenic zone in mesial temporal lobe epilepsy, particularly in combination with FDG-PET. Taken together, these findings indicate that qT2 is sensitive to mesial temporal tissue alterations, but that regional qT2 elevation should not by itself be equated with epileptogenicity.

On conventional MRI, the patient showed only a unilateral right-sided pattern, short of the bilateral mesial temporal T2/FLAIR abnormality that the Graus et al. (2016) criteria require for definite autoimmune LE (1). The presentation was thus best characterized as a limbic encephalitic syndrome formally fulfilling criteria for probable antibody-negative autoimmune encephalitis. Importantly, subsequent validation studies have shown that the criteria for definite autoimmune LE are highly specific but relatively insensitive, and that the requirement for bilateral conventional MRI abnormalities may limit classification of otherwise clinically compatible antibody-negative limbic cases (2, 18). In this context, the bilateral relaxation abnormalities observed in our patient should not be regarded as replacing the established imaging criterion, but rather as complementary evidence that quantitative MRI may detect mesial temporal tissue alterations beyond those visible on conventional imaging. Whether MR relaxometry can improve the detection of clinically relevant mesial temporal abnormalities in such radiologically borderline cases requires validation in larger cohorts.

Proton MRS provided complementary information on metabolic alterations within the mesial temporal regions. tNAA was reduced bilaterally, with a more pronounced reduction on the right side. Reduced NAA is associated with neuronal dysfunction or injury, which in the present clinical context may reflect, at least in part, inflammation-related neuronal involvement (9). However, NAA reduction is not specific for inflammation and may also occur in temporal lobe epilepsy (19, 20). Right-sided tCho was also lower than in the healthy reference subject (3.79 vs. 4.80 mmol/L; −21.0%), whereas left-sided tCho was comparable (4.46 vs. 4.70 mmol/L). No similarly pronounced difference was observed for the remaining quantified metabolite. A distinct but low-amplitude out-of-voxel artifact in the approximately 2.8–3.6 ppm range may have influenced quantification of metabolites with resonances within this spectral region, including tCho and tCr. Taken together, the spectroscopic findings support bilateral mesial temporal metabolic involvement with greater right-sided involvement, although they cannot distinguish inflammatory from seizure-related neuronal dysfunction (9).

Notably, the documented electroclinical seizure onset was confined to the right temporal lobe, whereas quantitative MRI and MRS demonstrated abnormalities extending bilaterally. Although contralateral subclinical or propagated epileptic activity cannot be excluded by scalp EEG, this spatial discrepancy makes it less straightforward to attribute all bilateral quantitative and metabolic alterations solely to the recorded right temporal seizure focus and supports tissue involvement beyond the electrophysiologically dominant side.

The serum anti-PDH antibody finding is noteworthy but requires cautious interpretation. Anti-PDH complex autoantibodies have recently been identified in a selected subset of patients with seizures and/or psychiatric symptoms who were negative for established antineuronal autoantibodies, with clinical features overlapping with those observed in our patient (21). Experimental studies demonstrated neuronal binding and reduced PDH activity under *in vitro* conditions, supporting the mechanistic plausibility of a potential functional role. However, their diagnostic and pathogenic significance in autoimmune encephalitis remains uncertain, and anti-PDH antibodies should currently be regarded as an associated immunological finding rather than a disease specific biomarker or established cause of autoimmune encephalitis.

This distinction is particularly relevant in the present case, as anti-PDH/antimitochondrial reactivity is not specific for autoimmune encephalitis and has also been reported in SSc and related connective tissue disease (22). Thus, serum anti-PDH positivity cannot be considered independent neuroimmunological evidence for autoimmune limbic encephalitis and may, at least in part, reflect the patient's underlying systemic autoimmune background. Central nervous system involvement is unusual in SSc and is more commonly discussed in the context of vasculopathy than direct parenchymal inflammation. Rare case reports, however, have described inflammatory central nervous system syndromes in SSc, including autoimmune LE (4). In our patient, SSc appeared clinically quiescent at follow-up, arguing against a simple parallel systemic flare. Nevertheless, the presence of SSc-associated anti-PDH positivity reflects a pronounced systemic autoimmune background. Accordingly, the diagnosis of a limbic encephalitic syndrome rests primarily on the clinical presentation and the concordant EEG and multimodal imaging findings rather than on anti-PDH positivity. While coincidental coexistence cannot be excluded, the case supports considering LE within a broader context of systemic immune dysregulation rather than as an entirely isolated neurological event.

The findings presented in this case are subject to several limitations. As a single-case observation, the study is inherently limited in its generalizability, and the small healthy reference samples for qT1 (*n* = 7) and qT2 (*n* = 5) limit the precision of the between-subject standard deviation estimates and consequently the stability of standardized effect sizes, particularly in regions with low reference variability. In addition, the MRS comparison was based on a single healthy reference subject, and a residual out-of-voxel artifact represents a potential source of bias in metabolite quantification. A further limitation is that quantitative MRI and MRS could not be repeated at follow-up, precluding assessment of longitudinal changes in the observed relaxation and metabolic abnormalities. Finally, the diagnostic and pathogenic significance of anti-PDH antibodies in autoimmune encephalitis remains uncertain.

## 5 Conclusion

In conclusion, this case of antibody-atypical LE in a patient with SSc illustrates how multimodal imaging with FDG-PET/CT-MRI fusion, MR relaxometry, and proton MRS can converge to support the diagnosis when routine neural-antibody testing is negative. In particular, MR relaxometry revealed bilateral, though non-uniformly lateralized, temporomesial abnormalities beyond the right-sided findings on conventional imaging alone, while proton MRS demonstrated a bilaterally reduced, right-accentuated tNAA signal. Together, these findings are compatible with tissue inflammation and neuronal dysfunction extending beyond the range of conventional imaging and highlight the potential value of quantitative imaging as complementary adjuncts to conventional imaging.

## Data Availability

The datasets presented in this article are not readily available because of ethical and privacy restrictions. Requests to access the datasets should be directed to the corresponding author.
